# Antibiotic prescribing patterns in pediatric patients using the WHO access, watch, reserve (AWaRe) classification at a quaternary hospital in Nampula, Mozambique

**DOI:** 10.1038/s41598-024-72349-4

**Published:** 2024-09-30

**Authors:** Sancho Pedro Xavier, Ageo Mario Cândido da Silva, Audêncio Victor

**Affiliations:** 1 Institute of Collective Health, Federal University of Mato Grosso, Av. Fernando Correa da Costa, 2367 - Bairro Boa Esperança, Cuiabá, Mato Grosso 78060-900 Brazil; 2 School of Public Health, University of São Paulo (USP), Avenida Doutor Arnaldo, 715, São Paulo, 01246904 Brazil

**Keywords:** Antibiotics Prescription, Pediatric Patients, WHO AWaRe classification, Mozambique, Paediatric research, Bacterial infection, Malaria, Antibiotics

## Abstract

Antibiotics are often prescribed inappropriately, either when they are not needed or with an unnecessarily broad spectrum of activity. This is a serious problem that can lead to the development of antimicrobial resistance (AMR). This study was conducted to assess the antibiotic prescribing pattern in pediatric patients hospitalized at a quaternary hospital in Nampula, Mozambique, using the WHO indicators and Framework as a reference. A retrospective study was conducted using secondary data obtained from medical records. The study population consisted of children aged 0–10 years who were hospitalized in a quaternary-level hospital ward in Nampula, Mozambique. The pattern of antibiotic prescriptions was assessed using indicators and the WHO classification of antibiotics into AWaRe categories. Descriptive statistics were applied. A total of 464 antibiotics were prescribed during the study. The age groups of 1–3 years and 28 days-12 months were prescribed more antibiotics. The most common antibiotics were ceftriaxone and crystallized penicillin, which were frequently prescribed for patients suffering from bronchopneumonia, gastroenteritis, and malaria. 74.8% of the antibiotics prescribed belonged to the Access group, while 23.7% belonged to the Watch group. There were no prescriptions of antibiotics from the Reserve group. The average number of antibiotics per prescription was 1.51 (SD ± 0.725). The percentage of antibiotic prescribing was 97.5%, with 96.20% by injection. All antibiotics prescribed were on the essential medicines list and prescribed by generic name. These results are concerning and highlight the urgency of strengthening antimicrobial optimization measures, as well as implementing the AWaRe framework in antibiotic prescribing as an essential strategy to combat AMR.

## Introduction

Antibiotic use in hospitals, particularly inappropriate use, has been a crucial factor in the global rise of antimicrobial resistance (AMR), a global public health problem affecting humans, animals, agriculture, and the environment^1,2^. AMR is increasingly being reported globally, associated with an estimated 4.95 million deaths in 2019, including 1.27 million deaths directly attributable to AMR^3,4^. It is particularly alarming in sub-Saharan Africa, where excessive and inappropriate use could lead to more than 10 million deaths annually by 2050 if not addressed^1,3^. Programs that provide antimicrobial optimization are necessary, and are considered an essential element in the global response to AMR^5^.

The World Health Organization (WHO) developed the Access, Watch, and Reserve (AWaRe) antibiotic classification framework in 2017, and revised it in 2019 and 2021, to guide the empirical treatment of common diseases in children and adults. This guide is essential for healthcare professionals around the world, aligning with the WHO Model Lists of Essential Medicines and optimizing the quality and quantity of antibiotic prescriptions, helping to combat global antimicrobial resistance^6–9^. The Access group comprises first- or second-line antibiotics for common empirical treatments and should be widely available. The Watch group includes antibiotics with a high risk of resistance, to be used only in specific indications. These need to be monitored and prioritized as targets for management programs. The Reserve group contains last-resort antibiotics, used only in special circumstances to preserve their efficacy^10^.

The WHO AWaRe classification is a crucial tool for monitoring antibiotic prescriptions and optimizing antimicrobial programs, helping in the fight against antimicrobial resistance (AMR). WHO has set a target that by 2023, at least 60% of antibiotics used nationally should be from the Access group^8^. By prioritizing responsible use, we seek to make treatments safer, contain AMR, and promote global health security^11^.

In 2018, a global study highlighted significant variations in antibiotic prescribing for children and neonates, ranging from 7.8% in China to 61.2% in Slovenia for children, and from 24.2% in China to 100% in Singapore for neonates. In addition, there were large regional discrepancies in antibiotic prescribing for lower respiratory tract infections and neonatal sepsis among WHO member countries^12^. Since the implementation of the AWaRe system, several regions have shown remarkable progress, especially in countries that have adopted antimicrobial optimization policies, including prescribing guidelines and surveillance systems to monitor antibiotic use and resistance. The guidance provided by AWaRe helps clinicians choose specific antibiotics for particular conditions^13,14^.

However, to the best of our knowledge, there are no published data in Mozambique to evaluate the pattern of antibiotic prescribing in pediatric patients according to the WHO AWaRe Framework. Therefore, we conducted this study to evaluate the pattern of antibiotic prescribing in hospitalized pediatric patients at a quaternary hospital in Nampula to identify opportunities for improvement and strengthen the evidence on antimicrobial optimization in hospitalized pediatric patients.

## Methods

### Study design

A retrospective study was conducted using secondary data obtained from medical records. The research focused on hospitalized pediatric patients at the largest hospital in the city of Nampula, Nampula Province, Mozambique. The hospital is a quaternary-level facility. Pediatrics I is a ward that treats patients with various pathologies, categorized into gastrointestinal, respiratory, and general diseases. This study is a continuation of a previous research project^1^. The research was conducted in 2020.

### Population and sample size

From January to July 2019, 1,745 patients, aged 1 to 120 months, were admitted to the hospital ward under study. For this research, a sample size of 315 patients was determined using the population proportion formula^15^, calculated through the Epi Info software^16^. The calculation was based on a 95% confidence level, a 50% expected frequency, and a 5% margin of error, with adjustments for the finite population.

To determine the sample size, we used the adjusted Cochran formula for finite populations:

$$n=\frac{p(1-p){Z}^{2}N}{{\text{d}}^{2}\left(N-1\right)+{Z}^{2}p(1-p)}$$

Given the population size (N = 1,745), a 95% confidence level (Z = 1.96), maximum variability (p = 0.5), and a 5% margin of error (d = 0.05), the sample size was calculated to be 315 patients. This approach is consistent with the guidelines for sample size determination outlined by Charan and Biswas^17^

### Data collection and study variables

We utilized the WHO's AWaRe classification^18^ and the WHO/INRUD Prescription Indicators to scrutinize antibiotic prescription in a detailed manner. The objective was to assess specific prescription patterns, providing insights into the effectiveness and appropriateness of global prescription practices in accordance with WHO guidelines. Data were collected between October 2019 and February 2020 under the supervision of the principal investigator through an instrument encompassing sociodemographic and clinical information of patients, along with details regarding antibiotics used during hospitalization (refer to the article^1^ for more details). In cases where necessary information was absent from medical records, patients were excluded and replaced randomly. Microbiological reasons for prescriptions were not identified in the medical records.

### Statistical analysis

The Statistical Package for Social Sciences (IBM SPSS Statistics V27.0) software was used to analyze the data. Descriptive statistics, including frequencies, percentages, means, and standard deviations (SD), were used to present the results.

### Ethics approval and consent to participate

The research protocol received approval from the Ethics Committee of Universidade Lúrio, in the Faculty of Health Sciences, Pharmacy Department. Subsequently, it was also approved by the Research Ethics Committee of Hospital Central de Nampula, following evaluation in the Nursing Department, where the data were collected.

Given the retrospective nature of the research, which involved the analysis of existing clinical records and medical prescriptions without direct interactions or experiments with human subjects, obtaining individual informed consent was deemed unnecessary and aligned with ethical guidelines. This decision was formally documented, including a waiver statement from the respective ethics committees (Universidade Lúrio, Faculty of Health Sciences, and Hospital Central de Nampula), reaffirming continuous adherence to ethical guidelines.

It is important to note that the study followed ethical standards, implementing measures to ensure the privacy and confidentiality of data collected from clinical processes and medical prescriptions, in accordance with the principles of the Helsinki Declaration.

## Results

The demographic and clinical characteristics of the patients can be found in the previous study, including the number of antibiotics per prescription, duration of hospitalization, and other features. In that study, 97.5% of hospitalized children received a prescription with at least one antibiotic, totaling 464 prescribed antibiotics^1^.

### Distribution of prescribed antibiotics according to age

As shown in Table 1, which presents the distribution of antibiotics according to age group, antibiotics were most prescribed for children aged 1 to 3 years (39.91%) and 28 days to 12 months (37.07%). Among children aged 1 to 3 years, the most prescribed antibiotics were metronidazole (44.44%), ampicillin (44.19%), ceftriaxone (41.05%), and gentamicin (40.38%). For children aged 28 days to 1 year, the most prescribed medications were sulfamethoxazole and trimethoprim (54.65%), crystalline penicillin (28.39%), ceftriaxone (34.74%), ampicillin (46.51%), and gentamicin (28.85%).

**Table 1:** Distribution of Antibiotics Prescribed According to Age.

Antibiotics prescribed	ATC Code	**Age**								Total
		28 days-12 months		1–3 years		4–5 years		6–10 years		
		n	%	n	%	n	%	N	%	n
Crystallized penicillin	J01CE01	44	28,39	62	40,0	20	12,90	29	18,71	155
Sulphonamides and trimethoprim	J01EE01	47	54,65	30	34,88	4	4,65	5	5,81	86
Ampicillin	J01CA01	20	46,51	19	44,19	3	6,98	1	2,33	43
Ceftriaxone	J01DD04	33	34,74	39	41,05	14	14,74	9	9,47	95
Ciprofloxacin	J01MA02	0	0	3	60,0	1	20,0	1	20,0	5
Metronidazole	P01AB01	1	11,11	4	44,44	1	11,11	3	33,33	9
Gentamicin	J01GB03	15	28,85	21	40,38	2	3,85	14	26,92	52
Vancomycin	J01XA01	3	60,0	2	40,0	0	0,0	0	0,0	5
Nystatin	D01AA01	5	71,43	2	28,57	0	0,0	0	0,0	7
Tetracycline	J01AA07	2	100,0	0	0,0	0	0,0	0	0,0	2
Azithromycin	J01FA10	2	40,0	2	40,0	1	20,0	0	0,0	5
**Total**		172	37,07	184	39,91	46	9,91	62	13,36	464

### Distribution of antibiotics according to specific disease

According to Table 2, the most prescribed antibiotics were crystalline penicillin (33.41%), sulfamethoxazole and trimethoprim (18.53%), ceftriaxone (18.53%), and gentamicin (11.21%). Crystalline penicillin was predominantly prescribed for patients suffering from bronchopneumonia (57; 56.4%), followed by febrile convulsions (20; 58.8%), malaria (19; 45.2%), and gastroenteritis (14; 7.0%). Sulfamethoxazole-trimethoprim was most frequently prescribed for patients suffering from gastroenteritis (80; 43.2%). Ceftriaxone was the primary choice for patients suffering from gastroenteritis (50; 27.0%), followed by malaria (12; 28.6%), febrile convulsions (6; 17.6%), and typhoid fever (5; 45.5%). Gentamicin was predominantly prescribed for patients suffering from bronchopneumonia (28; 27.7%), malaria (4; 9.5%), and febrile seizures (3; 8.8%).

**Table 2:** Distribution of Antibiotics by Specific Diseases.

Specific Diseases	Antibiotics prescribed										Total	
	CryPen (A)	STrim (A)	AMP (A)	CTR (W)	CIP (W)	AZM (W)	MET (A)	TCN (A)	VAN (W)	GEN (A)	NST (Un)	n
	n (%)	n (%)	n (%)	n (%)	n (%)	n (%)	n (%)	n (%)	n (%)	n (%)	n (%)	
Gastroenteritis (A09, K52)	14 (7,0)	80 (43,2)	25 (13,5)	50 (27,0)	2 (1,1)	5 (2,7)	5 (2,7)	1 (0,5)		2 (1,1)	2 (1,1)	186
Malaria (B50)	19 (45,2)	1 (2,4)	3 (7,1)	12 (28,6)	1 (2,4)		1 (2,4)			4 (9,5)	1 (2,4)	42
Febrile convulsions (R59.0)	20 (58,8)		2 (5,9)	6 (17,6)			2 (5,9)		1 (2,9)	3 (8,8)		34
Bronchopneumonia (J18.0)	57 (56,4)	3 (3,0)	5 (5,0)	4 (4,0)				1 (1,0)	1 (1,0)	28 (27,7)	2 (2,0)	101
Hydrocephalitis (G90)	1 (16,7)		1 (16,7)	2 (33,3)					1 (16,7)	1 (16,7)		6
Anemia (D53)	6 (50,0)	1 (8,3)		1 (8,3)					1 (8,3)	2 (16,7)	1 (8,3)	12
Pharingotonsillits (J06.8)	5 (71,4)			1 (14,3)						1 (14,3)		7
Typhoid fever (A01)	2 (18,2)			5 (45,5)	2 (18,2)					2 (18,2)		11
Burn (T29)	1 (100,0)											1
Sepsis (A41.9)	8 (50,0)		3 (18,8)	3 (18,8)						2 (12,5)		16
Epilepsy (G40.0)	4 (66,7)			1 (16,7)			1 (16,7)					6
Injury (T14.9)				2 (100,0)								2
Alergy (T78.4)	2 (100,0)											2
Asthma (J46)	4 (57,1)		1 (14,3)	1 (14,3)						1 (14,3)		7
Tonsilitis (J03)	1 (50,0)									1 (50,0)		2
Haemangioma (T18)			1 (33,3)	1 (33,3)						1 (33,3)		3
Neoplasm (D36, C80.9)	2 (100,0)											2
Pyoderma (L0.80)	2 (50,0)									2 (50,0)		4
Pains (R52)				1 (100,0)								1
Cerebral palsy (G80)	2 (50,0)			1 (25,0)						1 (25,0)		4
Heart disease (I25-I69)			2 (50,0)	1 (25,0)						1 (25,0)		4
Splenomegaly (R16, B54)	1 (100,0)											1
Dermatitis (L20-L30)				2 (100.0)								2
Meningoencephalitis (G04)	1 (20,0)			2 (40,0)					1 (20,0)		1 (20,0)	5
Pharingitis (J02, J31.2)	2 (66,7)	1 (33,3)										3
Laryngitis (J04, J37)	1 (100,0)											1
Total (n)	155 (33,41)	86 (18,53)	43 (9,27)	86 (18,53)	5 (1,08)	5 (1,08)	9 (1,94)	2 (0,43)	5 (1,08)	52 (11,21)	7 (1,51)	464

The blank spaces in the table indicate that no antibiotics were prescribed for the specific disease during the study period.

*CryPen* Crystallized penicillin, *Strim* Sulphonamides and trimethoprim, *AMP*  Ampicillin, *CTR* Ceftriaxone; CIP = Ciprofloxacin; *AZM* Azithromycin, *MET* Metronidazole, *TCN*  Tetracycline, *VAN* Vancomycin, *GEN* Gentamicin, *NST* Nystatin, *A* Access, *W*  Watch; *Un* Unclassified.

### Distribution of prescribed antibiotics according to WHO's AWaRe classification

As shown in Table 3, 74.8% of the antibiotics prescribed belonged to the Access group, while 23.7% belonged to the Watch group. There were no prescriptions for antibiotics from the Reserve group. Among the antibiotics in the Access group, crystalline penicillin (155 prescriptions), sulfamethoxazole and trimethoprim (86 prescriptions), and gentamicin (52 prescriptions) were the most common. In the Watch group, ceftriaxone was the most prescribed antibiotic (95 prescriptions).

**Table 3:** Distribution of Antibiotics Prescribed According to the WHO AWaRe Classification.

Antibiotics prescribed	WHO AWaRe classification (group)			Total
	Access	Watch	Unclassified	
Crystallized penicillin	155			155
Sulphonamides and trimethoprim	86			86
Ampicillin	43			43
Ceftriaxone		95		95
Ciprofloxacin		5		5
Azithromycin		5		5
Metronidazole	9			9
Tetracycline	2			2
Vancomycin		5		5
Gentamicin	52			52
Nystatin			7	7
Total	347 (74,78)	110 (23,71)	7 (1,51)	464

### WHO/INRUD core prescribing indicators

According to Table 4, the average number of antibiotics per prescription was 1.51 (SD ± 0.725). The maximum number of antibiotics per prescription was 4. The percentage of antibiotic prescriptions was 97.5%, with 96.20% by injectable route. All antibiotics prescribed were on the list of essential medicines.

**Table 4:** World Health Organization/ International Network of Rational Use of Drugs (INRUD) core prescribing indicators used to assess the study's prescriptions.

Parameter	Frequency (n)	Percentage (%)	Reference standards (%)^28^
Average number of drugs per encounter	1.52 (SD: 0.725)	NA	1.6 – 1.8
Encounters prescribed antibiotics	307	97,5^1^	20 – 26.8
Encounter prescribed injections	304	96.20	13.4–24.1
Antibiotics prescribed are generics	464	100	100
Antibiotics prescribed in the essential medicine list	464	100	100

*NA* Not available.

## Discussion

This study represents the first in Mozambique to assess the patterns of antibiotic use in hospitalized children in a quaternary care hospital, using the WHO AWaRe classification. The research findings show the worrying trend of inappropriate prescribing in the country. This puts an additional burden on patients, who are forced to bear the costs of this irrational prescribing.

Our study revealed a predominant prescription of antibiotics for children aged 1 to 3 years (39.91%) and those between 28 days and 12 months (37.07%). Among children aged 28 days to 1 year, the most prescribed drugs included sulfamethoxazole and trimethoprim, crystallized penicillin, ceftriaxone, ampicillin, and gentamicin. Conversely, for children aged 1 to 3 years, the most commonly prescribed antibiotics were metronidazole, ampicillin, ceftriaxone, and gentamicin. These findings align with a study in pediatric patients at a hospital in Ghana, which also emphasized the frequency of gentamicin and ampicillin. Furthermore, ceftriaxone, gentamicin, ampicillin, crystallized penicillin, and metronidazole emerged as the most prescribed antibiotics for both age groups^19^. In an Ethiopian study, a combination of ampicillin and gentamicin was prescribed for patients under one month of age, as well as for those between one month and 1 to 5 years of age. Notably, there was a high prevalence of ceftriaxone in children aged 1 month to 1 year^20^. These studies highlight similar prescribing patterns, influenced by specific contextual factors in sub-Saharan African countries.

Concerning the distribution of antibiotics based on specific diseases in this study, it was observed that crystallized penicillin was more frequently prescribed for bronchopneumonia, followed by febrile seizures, malaria, and gastroenteritis. Sulfamethoxazole and trimethoprim were the most commonly prescribed antibiotics for cases of gastroenteritis, while ceftriaxone was preferentially used to treat gastroenteritis, malaria, and febrile convulsions. Gentamicin was predominantly used in the treatment of bronchopneumonia and malaria. A previous study demonstrated that combinations like ceftriaxone and gentamicin, as well as ceftriaxone and crystallized penicillin, were the most frequently prescribed antibiotics for bronchopneumonia^19^. The dangers associated with the irrational use of antibiotics become apparent in the potential emergence of resistant bacteria^21^. Inappropriate use of antibiotics, such as using them to treat malaria or gastroenteritis, represents a significant concern. Malaria, for instance, is not caused by bacteria, and gastroenteritis, in most cases, does not necessitate antibiotic treatment. Since gastroenteritis is often viral in origin, the disease is usually self-limiting, irrespective of the causative pathogenic agent, and typically requires rehydration^22^.

Regarding the WHO AWaRe classification in our study, 74.8% of prescribed antibiotics fell into the Access group, with 23.7% in the Watch group; notably, there were no prescriptions from the Reserve group. The most commonly prescribed antibiotics in the Access group were crystallized penicillin (155 prescriptions), sulfamethoxazole and trimethoprim (86 prescriptions), and gentamicin (52 prescriptions). Within the Watch group, ceftriaxone was the most prescribed antibiotic, accounting for 95 prescriptions. When comparing our findings with a study from Okinawa, Japan, we observed similar trends, with a prevalence of antibiotics from the Access and Watch groups in both studies^23^. Another study found greater use of antibiotics from the Watch group^24^. Interestingly, a study in the Caribbean reported a high prevalence in the Access group (57.6 and 71.0%) with no prescriptions from the Reserve group^25^. Conversely, a study in Zambia found a high prevalence in the Watch group, particularly with ceftriaxone^26^. Similar findings were reported in research conducted in China, where the most prescribed group was Watch, with azithromycin and third-generation cephalosporins being the most common^27^. In Bangladesh, the most frequently prescribed groups were Watch (64.0%), Access (35.6%), and the Reserve (0.1%). These studies collectively highlight the ongoing challenges in antibiotic prescribing patterns across different regions. According to WHO recommendations, at least 60% of total antibiotic consumption should come from the Access group. Our study and others indicate variable and concerning adherence to this guideline, suggesting the need for continued efforts in antimicrobial stewardship to better align with global recommendations and optimize antibiotic use in both pediatric and adult practices.

Concerning the WHO/INRUD prescribing indicators, in this study, the average number of antibiotics per prescription was 1.51 (SD ± 0.725), with a maximum of 4 antibiotics per prescription. The percentage of antibiotic prescriptions was 97.5%, and 96.20% of these were administered through injection. Notably, all prescribed antibiotics were listed in the essential medicines list. However, apart from the average number of antibiotics per prescription, the use of the essential medicines list, and prescriptions by the generic name, other key indicators surpassed the WHO-recommended values, suggesting inappropriate prescribing practices in this study^28^. Comparable results were observed in studies conducted in Tanzania^29^, Pakistan^28^, and India^30^. Conversely, Saudi Arabia reported lower figures, with an average of 1.26 antibiotics per prescription and an antibiotic prescription rate of 17.6%. Additionally, injectable antibiotics constituted 15.2% of the antibiotic prescriptions^31^. These variations may stem from factors such as potential inflation of rates due to physician non-compliance with practice standards or a lack of hospital resources for confirming infections through cultures. Notably, in Saudi Arabia, widespread government support, fueled by robust economic revenues from the oil industry, enables physicians to request laboratory cultures and confirm suspected microbes before prescribing antibiotics^32^.

Given the escalating challenge of antimicrobial resistance, strengthening antimicrobial optimization under government guidance is crucial. The adoption of WHO AWaRe guidelines and indicators, including those from the World Health Organization/International Network of Rational Use of Drugs (INRUD), is strongly recommended at all levels of health units, serving as essential tools for effective antibiotic management. Integrating the AWaRe classification into antibiotic prescribing practices and conducting regular audits to ensure adherence to these guidelines are critical steps. Additionally, investing in laboratory infrastructure to support accurate and timely diagnostic testing will help reduce unnecessary antibiotic use by ensuring prescriptions are based on confirmed diagnoses. It is also vital to promote rational prescribing practices by educating healthcare providers about the risks of inappropriate antibiotic use and the importance of following evidence-based guidelines. Emphasizing the need to prescribe antibiotics only when necessary and based on appropriate indications is essential. These measures, supported by WHO and INRUD indicators, establish a solid foundation for monitoring, evaluating, and improving antibiotic use, playing a pivotal role in preserving the effectiveness of treatments within a constantly evolving landscape of resistance^27,33^.

The present study has some limitations. Firstly, it was conducted in a single quaternary care setting, so the results may not apply to other types of settings or populations. Secondly, being a retrospective study, there's a possibility that some prescriptions were omitted, even though methods were implemented to ensure sample representativeness. Additionally, certain information wasn't collected as the study relied on a review of medical records. Despite these limitations, the study offers valuable insights into the prescribing patterns of an important referral hospital in the region.

## Conclusion

The findings of this study reveal a significant divergence between antibiotic prescription practices in Mozambique and WHO recommendations, with a troubling trend of excessive and inappropriate prescribing. Notably, the most prescribed antibiotics were from the Access group, followed by those from the Watch group. This observation is alarming and underscores the urgent need to reinforce antimicrobial stewardship. It highlights the importance of adopting the AWaRe framework and WHO indicators to guide and improve antibiotic prescribing practices, thereby mitigating the risk of antimicrobial resistance.

## Data Availability

The datasets used and/or analyzed during the current study are available from the corresponding author on reasonable request.

## Abbreviations

AMR: Antimicrobial resistance
AWaRe: Access, watch and reserve
IBM: International business machines corporation
*INRUD*: International network of rational use of drugs
SPSS: Statistical package for the social sciences
SD: Standard deviation
WHO: World health organization
